# Inheritance and flexibility of cell polarity: a clue for understanding human brain development and evolution

**DOI:** 10.1242/dev.199417

**Published:** 2021-09-09

**Authors:** Nereo Kalebic, Takashi Namba

**Affiliations:** 1 Human Technopole, 20157 Milan, Italy; 2 Neuroscience Center, HiLIFE - Helsinki Institute of Life Science, University of Helsinki, 00290 Helsinki, Finland

**Keywords:** Brain development, Brain evolution, Cell polarity, Neural stem cell, Neuronal polarity

## Abstract

Cell polarity is fundamentally important for understanding brain development. Here, we hypothesize that the inheritance and flexibility of cell polarity during neocortex development could be implicated in neocortical evolutionary expansion. Molecular and morphological features of cell polarity may be inherited from one type of progenitor cell to the other and finally transmitted to neurons. Furthermore, key cell types, such as basal progenitors and neurons, exhibit a highly flexible polarity. We suggest that both inheritance and flexibility of cell polarity are implicated in the amplification of basal progenitors and tangential dispersion of neurons, which are key features of the evolutionary expansion of the neocortex.

## Abstract (precis)

**Summary:** We suggest that the inheritance and flexibility of cell polarity are implicated in the evolutionary expansion of the developing neocortex by promoting the amplification of neural progenitors and tangential migration of neurons.

## Introduction

Cell polarity is a major determinant in the development of various organs (Barnes and Polleux, 2009; Campanale et al., 2017; Namba et al., 2015; Suzuki and Ohno, 2006). In this Hypothesis, we focus on the role of cell polarity (i.e. molecular or morphological polarity; see Glossary, Box 1) during the development of the neocortex. The neocortex is the outer sheet of mammalian cerebrum and it is widely considered to be the key structure underlying remarkable cognitive abilities in humans. During mammalian evolution, prominently in the human lineage, the neocortex experienced an extraordinary increase in its size accompanied by morphological changes (Dehay et al., 2015; Pattabiraman et al., 2020; Rakic, 2009). This expansion is a consequence of the developmental increase in the number of neurons, which is due to the amplification of neural progenitors. Neocortical folding, a morphological hallmark of neocortex expansion, is primarily enabled by the tangential dispersion of newborn neurons (Dehay et al., 2015; Fernández et al., 2016; Rakic, 2009).

**Box 1. Glossary**

**Molecular polarity:** This is an asymmetric localization (A, left) or activity (A, right) of molecules within a cell (Campanale et al., 2017; Suzuki and Ohno, 2006). Such polarized localization or activity in neural progenitors allows for an asymmetric inheritance of fate determinants and therefore controls proliferation versus differentiation during development. In mature neurons, the molecular polarity has important roles for the neuronal functions and is evidenced when comparing the molecular footprint of axon versus dendrite. The polarization of neural progenitors and neurons is determined by both cell-intrinsic and cell-extrinsic molecular factors.

**Morphological polarity:** This refers to an asymmetry in cell shape that serves as a structural basis for a function (Campanale et al., 2017). This is best reflected in the example of a neuron that contains two distinct types of processes (dendrites and axon), which enable a unidirectional and coordinated transmission of information (B, left). A recognizable example of morphological polarity of neural progenitors is the presence or absence of apical and basal processes (Götz and Huttner, 2005), which is linked to the proliferative capacity of progenitors (Kalebic and Huttner, 2020) (B, center). Cells undergoing directional migration must have a front-rear polarity, which is characterized by a protruding front and, at the opposite side, a retracting trailing edge (Llense and Etienne-Manneville, 2015). Migrating immature neurons contain this type of polarity, which is defined by the leading (front) and trailing (rear) processes (B, right).

**Polarity inheritance:** Inheritance of cell polarity from a mother stem cell to one of its daughter cells is defined by a transmission of asymmetrically distributed morphological features (C, left; asterisk indicates basally-directed process; # indicates apically-directed process) and/or molecules (C, right). This can allow daughter cells to assume functional characteristics similar to those of the mother cell.

**Polarity flexibility:** Flexibility of cell polarity refers to dynamic processes of multiple and reversible changes in the molecular and morphological polarity (D). It can occur in either postmitotic cells or in the interphase of the cycling cells. Flexibility in cell polarity leads to diverse morphotypes of neural progenitor cells, which may serve as a basis of their high proliferative capacity.

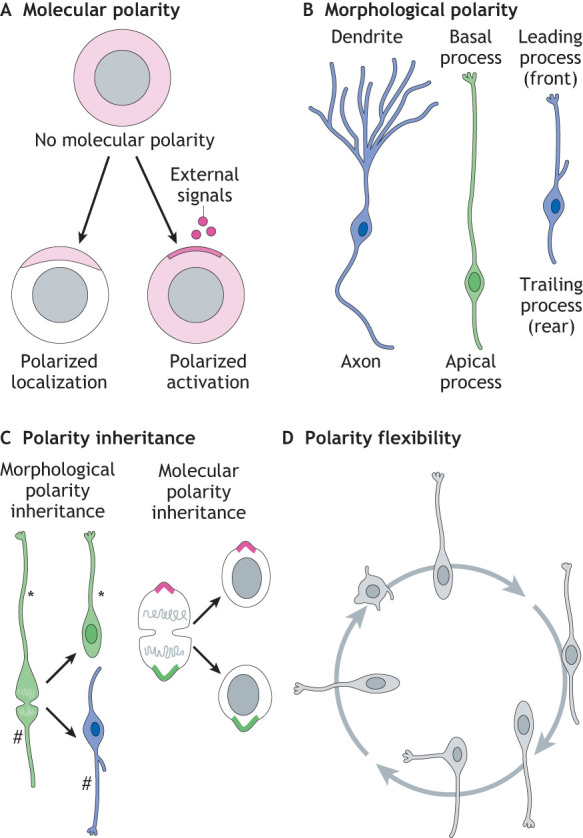

**Fig. 1.**
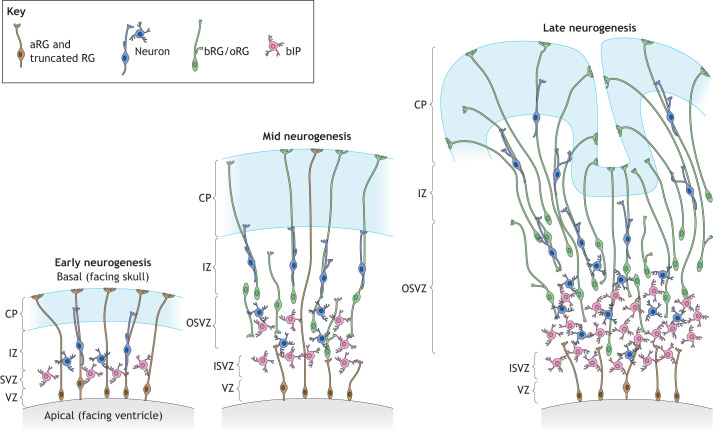
**Schematic of the neocortex development in gyrencephalic species.** During early neurogenesis (left) apical radial glia (aRG) are generating basal intermediate progenitors (bIP) that in turn produce neurons. At mid neurogenesis (middle), the subventricular zone (SVZ) is divided into two distinct layers (inner and outer SVZ; ISVZ and OSVZ, respectively) which are populated also by basal/outer radial glia (bRG/oRG). At late neurogenic stages (right), aRG become truncated and bRG become the key progenitor type, implicated in both generation of neurons and their tangential dispersion, contributing to neocortical expansion and folding. CP, cortical plate; IZ, intermediate zone; VZ, ventricular zone. Key applies to all subsequent figures.

The mammalian brain is built from a primordial neuroectoderm which develops into a pseudostratified neuroepithelium (Taverna et al., 2014). During early embryonic/fetal development, the neocortex exhibits apicobasal polarity characteristic of epithelial tissues, with the apical side facing the lumen of the lateral ventricles of the cerebrum and the basal side facing the skull (Fig. 1). At the onset of neurogenesis, neuroepithelial cells generate various types of neural progenitor cells that in turn produce neurons (Taverna et al., 2014). Both neuroepithelial cells and their immediate progeny, apical radial glia (aRG), show clear apicobasal polarity, which is best reflected in their morphology; they are elongated cells with an apical and a basal process that together enable the cell to maintain contact with both poles of the tissue (Götz and Huttner, 2005). The final output of neocortical neurogenesis, projection neurons, are par excellence polarized cells with a well-studied axon-dendrite polarity that is fundamental for their function (Barnes and Polleux, 2009; Namba et al., 2015; Tahirovic and Bradke, 2009). The existence and the importance of cell polarity both before and after neurogenesis are well-described, but many questions still remain unanswered.

Here, we provide a unifying theoretical framework and propose that, during neocortical neurogenesis, cell polarity can be inherited between different cell types and highly flexible (see Glossary, Box 1) within specific cell types in species with an expanded and folded neocortex, such as human. We propose that the inheritance and flexibility of cell polarity promote two fundamental features of neocortical expansion during human evolution: amplification of neural progenitors and tangential dispersion of neurons. Tight spatio-temporal regulation of cell polarity inheritance and flexibility in the developing neocortex could be crucial for understanding how our neocortex has developed and evolved.

## Inheritance of cell polarity in neural progenitors

Inheritance of cell polarity (see Glossary, Box 1) in neural progenitors is significant for the evolutionary expansion of the neocortex as it is often linked to the inheritance of a high proliferative capacity. For example, the inheritance of cell polarity from a mother aRG has an instrumental role in maintaining a high proliferative capacity of the daughter progenitor, thereby contributing to increased neuron production (Matsuzaki and Shitamukai, 2015; Taverna et al., 2014). Although it has already been described in the mammalian neocortex that cell polarity can be inherited between a mother aRG and a daughter aRG (Matsuzaki and Shitamukai, 2015), we propose that cell polarity can be inherited between other progenitor types. Notably, inheritance of cell polarity between basal progenitors (BPs) could be particularly relevant for the evolutionary expansion of the neocortex (see below).

### Apical progenitors

**Fig. 2.**
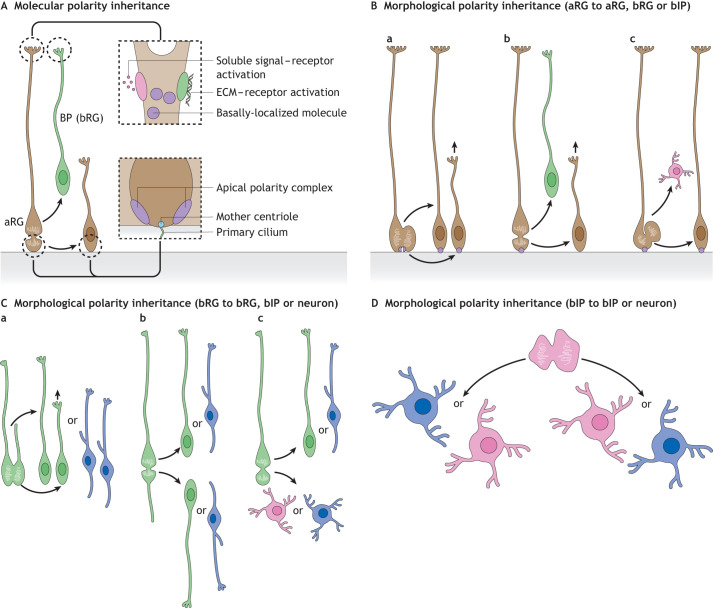
**Inheritance of cell polarity and cell fate.** (A) Molecular polarity can be passed on from mother cell to daughter cell, exemplified here by aRG and bRG. The daughter cell inheriting the basal process can also inherit the basally-localized molecules, respond to local soluble signals and extracellular matrix (ECM). The daughter cells inheriting the apical domain will receive the apical polarity complex and the mother centriole with the primary cilium. (B) Different modes of aRG division (a, vertical; b, horizontal; c, oblique) can result in distinct fates of daughter cells (a, two aRG; b, aRG and bRG; c, aRG and bIP). The apical complex is shown in purple, and straight black arrows denote process growth. (C) Cell divisions of various bRG morphotypes (a, bRG with a bifurcated basal process; b, bipolar bRG; c, bRG with a basal process) can result in distinct fates of daughter cells: two BPs, a BP and a neuron or two neurons. Straight black arrow denotes process growth. (D) bIPs in primates can undergo several rounds of proliferative division before generating neurons.

aRG are the first type of neural progenitors to emerge during development (Taverna et al., 2014). They have two principal functions: production of other neural cells (progenitors and neurons) and generation of a scaffold that supports neuronal migration. aRG contain many typical features of apicobasal polarity, such as the presence of the apical and basal processes, and an apical domain consisting of adherens junctions, apical polarity complex proteins, apical localization of the centrosome and localization of the Golgi apparatus in the apical process (Rakic, 2003a,b; Taverna et al., 2014, 2016). Classical work on aRG cell division in rodents states that this cell polarity is a key structural determinant for equal versus unequal distribution of cellular components, and thus symmetric versus asymmetric cell division (Arai and Taverna, 2017; Lu et al., 2000; Matsuzaki and Shitamukai, 2015; Taverna et al., 2014) (Fig. 2A). There are two different aspects to be considered when discussing the aRG division: the inheritance of the apical components and the inheritance of the basal process. The apical plasma membrane forms the apical surface facing the ventricle and bears the primary cilium (Taverna et al., 2014). During aRG division, the apical plasma membrane can either be bisected, when both daughter cells assume the aRG identity (Fig. 2B), or it can be bypassed by the cleavage furrow, with the daughter cell that inherits the apical plasma membrane maintaining the aRG identity and the other daughter cell delaminating and typically becoming a BP (Matsuzaki and Shitamukai, 2015; Taverna et al., 2014) (Fig. 2A,B).

The location of the primary cilium itself has also been suggested to play a role in determining cell fate in mouse because the daughter cell fated to undergo delamination displays a basolateral (instead of apical) cilium (Wilsch-Bräuninger et al., 2012). Linked to the primary cilium is the centrosome, the centrioles of which are differentially inherited by daughter cells. The centrosome containing the older centriole, the so-called mother centriole, is preferentially inherited by the daughter cell that remains an aRG, suggesting a role of the centrosome in cell fate and proliferative capacity of daughter cells (Paridaen et al., 2013; Wang et al., 2009). Moreover, the ciliary membrane tends to be inherited by the proliferative daughter cell (Paridaen et al., 2013). The inheritance of the aRG basal process is also associated with the maintenance of proliferative capacity, as it enables contact with the basal lamina and the pro-proliferative extrinsic signals present there (Girós et al., 2007). The molecular mechanisms underlying the contribution of basal process inheritance in determining the daughter cell fate and influencing the proliferative capacity of aRG have recently been tackled (Nakagawa et al., 2019; Okamoto et al., 2013; Pilaz et al., 2016; Tsunekawa et al., 2012).

### Basal progenitors

BPs are generated by aRG as they lose apical contact with the ventricle and migrate to a more basal germinal layer called the subventricular zone (SVZ), where they proliferate to generate other BPs and neurons (Haubensak et al., 2004; Miyata et al., 2004; Noctor et al., 2004) (Fig. 1). Mammalian BPs exhibit a striking diversity of cell polarities (Betizeau et al., 2013; Kalebic et al., 2019; Kalebic and Huttner, 2020; Reillo et al., 2017) and can broadly be divided into two subtypes: basal, or outer, radial glia (bRG or oRG) and basal intermediate progenitors (bIPs) (Fernández et al., 2016; Namba and Huttner, 2017). In species with a small and smooth neocortex, such as mouse, BPs have a very limited proliferative capacity, typically dividing only once to generate two neurons, whereas in species with an expanded and folded neocortex (such as humans, ferrets or macaques) BPs are highly proliferative and can undergo many rounds of cell division before they consumptively divide and finally generate neurons or glia (Betizeau et al., 2013; Hansen et al., 2010; Kalebic et al., 2017; Lui et al., 2011). Furthermore, the human, ferret and macaque neocortex contains a larger proportion of bRG among BPs (∼50%) (Betizeau et al., 2013; Fietz et al., 2010; Kalebic et al., 2019; Reillo et al., 2011) compared with mouse, in which bRG generally comprise up to 10% of BPs (Kalebic et al., 2019; Shitamukai et al., 2011; Wang et al., 2011). Together, this has led to the idea that bRG are the key cell type for understanding the evolutionary expansion of the mammalian neocortex (Borrell and Reillo, 2012; Fietz and Huttner, 2011; Lui et al., 2011).

#### Basal radial glia

Newborn bRG can arise upon horizontal cell division of aRG (LaMonica et al., 2013; Martínez-Martínez et al., 2016; Shitamukai et al., 2011), by inheriting the basal process of the mother cell, but not the apical plasma membrane (Fig. 2B). Like aRG, bRG also have two distinct functions: the generation of other neural cells and providing scaffold for neuronal migration (Kalebic and Huttner, 2020). bRG move their cell body to the SVZ where they undergo a process called ‘mitotic somal translocation’, which describes the migration of the soma with the cell nucleus to a basal position just before mitosis (Hansen et al., 2010). Therefore, bRG show polarized morphology at mitosis, similar to the apicobasal polarity of aRG (Fietz et al., 2010). However, they lack the apical junctional complex and the contact with the ventricle, but often retain the basal process in contact with the basal lamina. Owing to such characteristics, we refer to the bRG polarity at mitosis as a ‘pseudoapicobasal’. Interestingly, the majority of primate bRG divide horizontally, with the daughter cell that inherits the basal process becoming a bRG and the other daughter cell typically becoming either a bIP, bRG or neuron (Betizeau et al., 2013; LaMonica et al., 2013) (Fig. 2C).

#### Basal intermediate progenitors

bIPs can be generated by aRG (Fig. 2B), bRG (Fig. 2C) or other bIPs (Fig. 2D). When generated by aRG, newborn bIPs lose contact with both the ventricle and the pia and migrate to the SVZ. During interphase they exhibit a multipolar morphology, whereas in mitosis they generally become nonpolar (Haubensak et al., 2004; Miyata et al., 2004; Noctor et al., 2004; Wu et al., 2005) (Fig. 2D). As they do not inherit any features of morphological polarity from their mother radial glia, nor show any polarized distribution of organelles (Taverna et al., 2016) or molecules, their generation resembles a classical epithelial-to-mesenchymal transition (Itoh et al., 2013). Their proliferative capacity is greater in species with an expanded neocortex, which is likely linked to their ability to grow additional cell processes (Kalebic et al., 2019).

In conclusion, morphological polarity can be inherited between different progenitors: from mother aRG to daughter aRG or bRG and from mother bRG to daughter bRG. This inheritance can be associated with the inheritance of the proliferative capacity and/or cell fate.

## Flexibility of cell polarity in basal radial glia

Here, we propose that the flexibility of cell polarity (see Glossary, Box 1) is the second key feature underlying the neocortical expansion. We put forward that an increase in the flexibility of cell polarity leads to an increase in the cell's proliferative capacity.

**Fig. 3.**
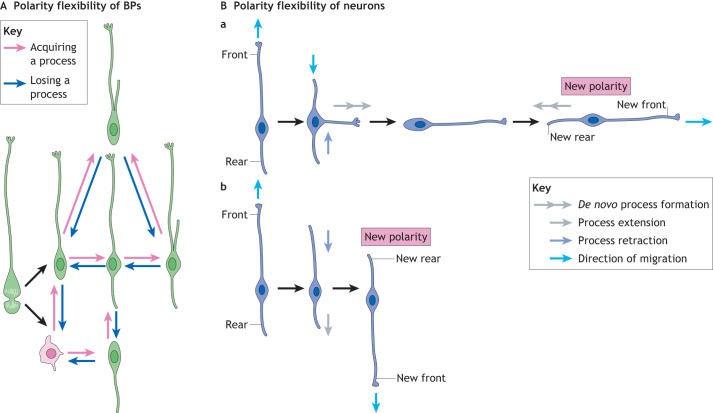
**Flexibility of cell polarity.** (A) Basal progenitors (BPs) show a high flexibility in their morphological polarity with the ability to acquire and lose their processes. BP morphologies include multipolar cells, bIPs and a subset of transient bRG (pink), along with five different radial morphotypes (green) that can contain up to two basal processes and an apically-directed process. (B) Migrating neurons show polarity flexibility as they generate *de novo* processes and retract or extend existing processes during tangential dispersion. There are two ways new polarity can be established: (a) a process formed *de novo* at a different part of the cell body becomes the new leading process. The old leading and trailing processes are eventually retracted, and a new trailing process arises opposite to the new leading process. (b) The trailing process extends and becomes the new leading process, resulting in the reversal of polarity. Straight light blue arrow denotes direction of migration.

Indeed, bRG in species with an expanded neocortex, such as human, ferret and macaque, show a high proliferative capacity and exhibit a high degree of flexibility in their cell polarity. Such flexibility is reflected in the variety of bRG morphotypes present in those species (Kalebic and Huttner, 2020), where ‘morphotype’ refers to a group of cells belonging to the same cell type that share distinct morphological characteristics (Fig. 3A). The originally described morphotype of a bRG is a monopolar cell with a long basal process that allows contact with the basal lamina (Fietz et al., 2010; Hansen et al., 2010; Reillo et al., 2011). However, bRG with a (short) apically-directed process that never reaches the apical surface, and the morphologically bipolar bRG with both the basal and the apically-directed process, have been detected in various mammalian species, such as human, ferret, mouse or macaque (Betizeau et al., 2013; Kalebic et al., 2019; Pilz et al., 2013; Reillo et al., 2017; Wong et al., 2015). Furthermore, in the developing macaque neocortex during interphase, bRG readily transition between morphotypes that show either an apical and/or a basal process and stages with no processes, known as transient bRG (Betizeau et al., 2013) (Fig. 3A).

In addition to different morphotypes, bRG in species with an expanded neocortex exhibit a high degree of flexibility in cell polarity after divison. For example, for a bRG daughter cell without the basal process to acquire bRG morphology, it needs to regrow its basal process (Betizeau et al., 2013), which enables the cell to adapt its morphology and thereby to maintain a high proliferative capacity away from the ventricle (Fig. 3A). Alternatively, instead of a daughter bRG regrowing the basal process, the mother bRG could potentially split its own basal process between the two daughter cells (Kalebic et al., 2019), similar to what has been observed in mouse neuroepithelial cells (Kosodo et al., 2008). The recent discovery of bRG with two basal processes or a single bifurcated basal process supports the idea that each daughter cell could inherit one basal process and thereby maintain contact with the basal lamina and a high proliferative capacity (Kalebic et al., 2019; Kalebic and Huttner, 2020) (Figs 2C and 3A).

Hence, bRG show extraordinary flexibility in their morphological cell polarity, with a particular heterogeneity of morphotypes found in species with an expanded neocortex (Betizeau et al., 2013; Kalebic et al., 2019; Kalebic and Huttner, 2020; Reillo et al., 2017) (Fig. 3A). It is still unclear what determines such flexibility and its relevance for the two distinct bRG functions: neuronal production and support for neuronal migration (Kalebic and Huttner, 2020). As bRG are potentially the greatest source of neurons in human developing neocortex, maintaining their proliferative capacity is crucial for the production of this enormous number of neurons. In accordance with this, it has been shown that both the number of bRG processes and the bRG proliferative capacity are greater in species with an expanded neocortex (i.e. humans, macaques), indicating that the number of cell processes can be a determinant of the bRG proliferative capacity (Kalebic and Huttner, 2020) (Table 1). For example, time-lapse imaging in the macaque neocortex revealed that those bRG that contain more processes are more proliferative and generate more neurons (Betizeau et al., 2013; Dehay et al., 2015). Furthermore, a reduction of bRG processes in human fetal neocortex leads to a reduction in bRG proliferative capacity, showing a causative relationship (Kalebic et al., 2019). Migrating neurons use the basal processes of both aRG and bRG to reach their final destination in the cortical plate (Rakic, 1972). Interestingly, at mid and late stages of human neocortex development only bRG fibers are used, because human aRG contain only truncated basal processes (Nowakowski et al., 2016) (Fig. 1A). Flexibility of bRG polarity, which is manifested in dynamic changes of bRG radial processes, leads to migrating neurons switching from one radial process to another and, thereby, cell polarity of bRG promotes tangential dispersion of neurons, which is a key feature of the development of an expanded neocortex (Fig. 1).

**Table 1.**
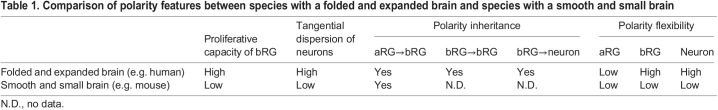
Comparison of polarity features between species with a folded and expanded brain and species with a smooth and small brain

In conclusion, bRG potentially inherit polarity from their mother cells and show a range of flexibility in cell polarity (Fig. 3A) that is likely linked to their functions (Table 1). Therefore, we propose that the flexibility of bRG polarity, exemplified here through the changes in morphology, could be one of the driving forces for the evolutionary expansion of the human neocortex.

## Inheritance and flexibility of cell polarity in neurons

Neurons are basic units of the nervous system and are responsible for the coordinated transmission of information. This transmission is unidirectional and is enabled by the axon-dendrite polarity of the neuron. There is a lot of diversity in how neurons are born; the majority of mammalian neocortical neurons are generated by BPs (both bIPs and bRG; Fig. 2), whereas only a minority are produced directly by aRG.

### Types of cell polarity in neurons

Newly generated neurons have two types of polarity: (1) front-rear polarity, which is required for their migration, and (2) axon-dendrite polarity, which is a prerequisite for their function (Barnes and Polleux, 2009; Namba et al., 2015; Tahirovic and Bradke, 2009).

#### Front-rear polarity

During development, an immature neuron establishes front-rear polarity, which is essential for directional migration (Buchsbaum and Cappello, 2019; Evsyukova et al., 2013; Kawauchi, 2015; Silva et al., 2019). Front-rear polarity is defined by the presence of a leading process (front) and a trailing process (rear), and determines the direction of cell movement. Depending on the morphotype of their mother progenitor cell, brain region and mammalian species, migrating neurons show a certain degree of flexibility in their front-rear polarity (Cortay et al., 2020; Gertz and Kriegstein, 2015; Martínez-Martínez et al., 2019; Namba et al., 2019) (Fig. 3B).

#### Axon-dendrite polarity

The leading process and the trailing process of a migrating neuron give rise to two types of processes in the mature neuron: dendrites and the axon, respectively (Namba et al., 2015). The composition of proteins and organelles in mature neurons differ between axon and dendrites, translating into both molecular and morphological differences. Thick and short dendrites serve to receive chemical signals from other cells through their neurotransmitter receptors. In contrast, typically thin and long axons at their terminals contain synaptic vesicles required for synaptic transmission to other cells. Thus, mature neurons are both molecularly and morphologically polarized, which underlies their function (Barnes and Polleux, 2009; Caceres et al., 1986; Cooper, 2014; Namba et al., 2015; Tahirovic and Bradke, 2009). However, some migrating neurons exhibit a flexibility in the front-rear polarity; that is, repeated extension and retraction of the leading and trailing processes. In these neurons, the fates of the leading process and the trailing process are not determined, thus the front-rear polarity cannot be considered as the axon-dendrite polarity.

### Modes of neuron polarization

**Fig. 4.**
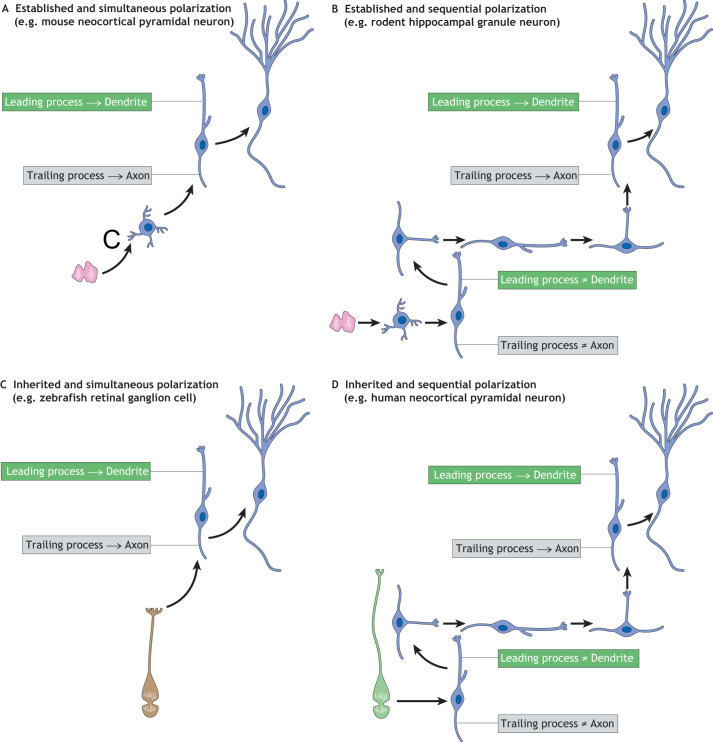
**Four different modes of neuronal polarization.** (A-D) Four different modes of neuronal polarization based on the timing of the front-rear and axon-dendrite polarity determinations. If the leading process and the trailing process will give rise to dendrites and an axon, respectively, the front-rear polarity can be considered equal to the axon-dendrite polarity. However, if neurons exhibit a flexibility in the front-rear polarity during migration, the fates of the leading process and the trailing process are not determined and hence the front-rear polarity is not equivalent to the axon-dendrite polarity. (A) Established and simultaneous polarization (e.g. mouse neocortical pyramidal neurons). (B) Established and sequential polarization (e.g. rodent hippocampal granule neurons). (C) Inherited and simultaneous polarization (e.g. zebrafish retinal ganglion cells). (D) Inherited and sequential polarization (e.g. pyramidal neurons in gyrencephalic neocortices, such as human).

We next bring forward the idea that the inheritance of cell polarity from bRG to neuron (Fig. 2C) allows the newborn neuron to acquire front-rear bipolar morphology independently, before the establishment of the axon-dendrite polarity. This, in turn, primes the neuron to exhibit the flexibility of front-rear polarity. Therefore, the inheritance of cell polarity from bRG to neuron facilitates the flexibility of the cell polarity of the daughter neuron. In relation to these ideas, we propose that newly-generated neurons can undergo four different modes of polarization: (1) establishing *de novo* front-rear and axon-dendrite polarities simultaneously (termed ‘established and simultaneous polarization’); (2) establishing the front-rear polarity first, and subsequently developing the axon-dendrite polarity (termed ‘established and sequential polarization’); (3) inheriting mother progenitor-cell polarity that simultaneously transforms into the front-rear and axon-dendrite polarities (termed ‘inherited and simultaneous polarization’); and (4) inheriting mother progenitor-cell polarity that transforms first into front-rear polarity and subsequently into the axon-dendrite polarity (termed ‘inherited and sequential polarization’) (summarized in Fig. 4).

#### Established and simultaneous polarization

The vast majority of neocortical neurons are generated from BPs (Fernández et al., 2016; Kriegstein et al., 2006; Namba and Huttner, 2017). When generated from non-polarized bIPs (for example, in the embryonic mouse neocortex), just after the cell division neurons exhibit a multipolar morphology (i.e. they extend multiple short processes) (Namba et al., 2015; Tabata and Nakajima, 2003). These processes undergo repeated extension and retraction until one of them starts to elongate continuously to become the trailing process (Namba et al., 2014). Thereafter, another process becomes the leading process and the multipolar neuron finally transforms into a bipolar neuron, which undergoes directional migration and thereby possesses front-rear polarity (Buchsbaum and Cappello, 2019; Evsyukova et al., 2013; Kawauchi, 2015; Silva et al., 2019). The identities of the leading (front) and trailing (rear) processes are fixed and they finally transform into dendrites and axon, respectively. Thus, the eventual axon-dendrite polarization of bipolar neurons occurs concomitantly with the onset of the front-rear polarity. Therefore, those two different polarities are established simultaneously when pyramidal neurons are generated from non-polarized bIPs, notably in embryonic mouse neocortex (Namba et al., 2015) (Fig. 4A).

#### Established and sequential polarization

An example of established and sequential polarization is observed in the rodent neonatal hippocampus (Namba et al., 2019). The newborn hippocampal granule neurons generated from bIPs exhibit a bipolar morphology (Namba et al., 2005, 2011), but the identities of their leading and trailing processes are flexible (Namba et al., 2019; Seki et al., 2007; Wang et al., 2018). For example, bipolar neurons often retract the current leading process and simultaneously extend a new leading process from another part of the cell body (Figs 3B and 4B). Concomitantly, the old trailing process is retracted and the new trailing process emerges opposite to the new leading process (Namba et al., 2019). Due to these morphological changes, the bipolar neurons are able to change their direction of movement dramatically, sometimes even towards the opposite direction. Only after the bipolar neurons fix the direction of their migration do they finally determine the identities of their processes and the leading and trailing processes become dendrite and axon, respectively (Fig. 4B). Therefore, in the rodent hippocampus, the bipolar neurons undergo two polarization events sequentially (Namba et al., 2019). As we discuss below, the sequential polarization could be beneficial for neurons to distribute in the tangential axis.

#### Inherited and simultaneous polarization

Polarized progenitor cells can transmit their cell polarity to the differentiated daughter cells (Namba et al., 2015). For example, the neuroepithelial cells in the developing vertebrate retina can transmit their apicobasal polarity to their daughter neurons, the retinal ganglion cells (Randlett et al., 2011). During their migration, these neurons maintain the polarity inherited from the mother cells by keeping physical contact with the basal lamina via its basally-directed process. This basal process eventually develops into an axon, whereas the dendrites emerge from the apical side of the neuron. Therefore, the neuron does not need to establish its polarity *de novo*, but the apicobasal polarity of the mother cells transforms simultaneously into both the front-rear and the axon-dendrite polarity (Fig. 4C).

#### Inherited and sequential polarization

In some gyrencephalic species, the newborn pyramidal neurons derived from the polarized bRG can inherit the cell polarity of the mother cell (Cortay et al., 2020; Gertz and Kriegstein, 2015; Martínez-Martínez et al., 2019) (Fig. 2C; Table 1). The inherited cell polarity transforms into the front-rear polarity of migrating newborn neurons. Importantly, such inheritance allows newborn neurons to possess the front-rear polarity without determining their axon-dendrite polarity (Fig. 4D). This in turn is the major prerequisite for maintaining flexibility of cell polarity during tangential migration (discussed below). Indeed, in gyrencephalic species, the majority of bipolar neurons show a striking flexibility in their front-rear polarity that consists of repeated retraction and *de novo* extension of their cell processes (Gertz and Kriegstein, 2015). This allows the newborn neurons to repeatedly change the direction of their migration, which in turn enables tangential dispersion followed by gyrus formation (Cortay et al., 2020; Gertz and Kriegstein, 2015; Martínez-Martínez et al., 2019). As to the axon-dendrite polarity, it is determined only after fixing the direction of the migration.

### Tangential migration of neurons as a result of flexibility in neuronal polarity

Neurons can inherit or not the cell polarity from their mother cells and they can exhibit different degrees of flexibility in establishing their front-rear polarity for migration. Neurons showing less flexibility migrate relatively straight along the radial axis. In contrast, neurons with a higher degree of flexibility in front-rear polarity migrate more tangentially, which might contribute to the development of the laterally expanded and folded brain (Fig. 1; Table 1) (Borrell and Götz, 2014; Buchsbaum and Cappello, 2019; Molnár et al., 2019). Two factors contribute to the tangential migration and dispersion of neocortical neurons.

First, the basal processes of aRG and bRG form a curved scaffold that supports neuronal dispersion (Borrell and Reillo, 2012; Fietz and Huttner, 2011; Lui et al., 2011; Rakic, 2009). In the mouse developing dorsolateral neocortex, in which the basal processes of aRG align perpendicular to the apical and basal surfaces, the neurons migrate radially to the cortical plate and show very limited tangential dispersion. In contrast, in the gyrus of the human developing neocortex, the bRG extend their basal processes in a fan-shaped manner and the neurons disperse more tangentially (Fig. 1).

The second factor for achieving tangential dispersion is an endogenous feature of a neuron, which is the ability to change direction during migration (Fig. 3B). As an adaptation to the flexibility of bRG morphology, some neocortical neurons can change their direction of migration, as reported in the ferret (Gertz and Kriegstein, 2015; Martínez-Martínez et al., 2019) and macaque (Cortay et al., 2020) developing neocortex (see above; Fig. 4D). The flexibility in the front-rear polarity has not only been found in the developing gyrenecephalic neocortex, but also in those brain regions of lissencephalic species that exhibit folding, such as hippocampal dentate gyrus (see above; Fig. 4B) (Namba et al., 2019). In addition, GABAergic interneurons are known to exhibit high degree of flexibility in their front-rear polarity to disperse tangentially within the neocortex (Tanaka et al., 2009). Therefore, we propound the idea that the flexibility of front-rear neuronal polarity, strongly associated with their migration in a gyrencephalic cortex, is a crucial factor enabling the tangential dispersion and is another driving force for the evolutionary expansion of the neocortex.

## Mechanisms of molecular and morphological polarity

In the sections above, we explored the inheritance and flexibility in neuronal progenitors and neurons. Here, we discuss how such molecular and morphological polarity can be achieved.

### Molecular polarity

#### Cell-intrinsic factors

The major way of establishing and maintaining molecular polarity is by polarized localization of proteins (see Glossary, Box 1; Fig. 2A) (Arai and Taverna, 2017; Barnes and Polleux, 2009; Hansen et al., 2017; Namba et al., 2015; Tahirovic and Bradke, 2009). A classical example of such localization in both aRG and neurons is the Par complex consisting of Par3, Par6 and aPKC (Namba et al., 2015). In aRG, the Par complex is involved in maintaining polarity on the apical side (Bultje et al., 2009; Hansen et al., 2017; Lui et al., 2011). In neurons it accumulates at the tip of the growing process and enables that process to grow faster and become longer than others, thereby inducing neuronal polarization (Barnes and Polleux, 2009; Namba et al., 2015; Tahirovic and Bradke, 2009). Interestingly, Par3 protein has not been found in bRG somata (Fietz et al., 2010), but its mRNA could be easily detected in the mouse bRG (Florio et al., 2015), suggesting that the Par complex could be localized in specific subcellular domains of bRG, such as basal process, where it could act to induce basal process growth.

Furthermore, the inheritance of the apical domain, and hence the Par complex, enables maintenance of the proliferative capacity of daughter aRG (Bultje et al., 2009). Par complex regulates the activity of Notch signaling through interaction between Par3 and Numb (Bultje et al., 2009), which modulates the plasma membrane localization of Notch (Kandachar and Roegiers, 2012). As activity of Numb is inhibited by aPKC-mediated phosphorylation (Nishimura and Kaibuchi, 2007), aRG that inherited the Par complex likely enrich Notch on the plasma membrane. This in turn leads to a higher Notch activity that enables those aRG to maintain their ‘stemness’. It is, therefore, tempting to speculate that, should Par complex be present also in bRG, it could play a role in the inheritance of molecular polarity between mother bRG and daughter bRG and in maintenance of the bRG proliferative capacity.

Other molecules potentially involved in inheritance of molecular polarity between aRG and bRG are the proteins and mRNAs present in the basal process itself, as well as in the basal endfoot. A seminal example is the localization of *Ccnd2* mRNA in the basal endfoot (Tsunekawa et al., 2012), which could be interpreted as a means of molecular polarity that allows the daughter cell with the inherited basal process to continue proliferating. In addition, various RNA-binding proteins such as Stau2, which is required for asymmetric cell division (Kusek et al., 2012), have been detected in the basal endfoot (Pilaz et al., 2016), suggesting a general mechanism that controls molecular polarity at the basal side.

It is currently unknown how features of molecular polarity could be inherited by the daughter neurons from their mother bRG. To explore such a scenario, one can examine a simpler model, the mammalian peripheral nervous system, in which neural crest cells generate dorsal root ganglion neurons. These progenitors lose their morphological polarity before the cell division, but their daughter neurons are able to extend a new process from the septin-enriched domain, which was inherited from the mother progenitor (Boubakar et al., 2017), thus constituting an example of inheritance of molecular polarity.

In addition to proteins with a polarized localization, local activation of ubiquitously distributed proteins has an important role for cell polarity in both progenitors and neurons (see Glossary, Box 1). A prominent role is exerted by cytoskeleton modifiers and molecules that link the cytoskeleton to the plasma membrane. These include small GTPases, such as Rac1 (Kawauchi et al., 2003; Namba et al., 2014; Tahirovic et al., 2010), Cdc42 (Garvalov et al., 2007; Yokota et al., 2010) and RhoA (Cappello et al., 2012; Xu et al., 2015), as well as MARCKS (Brudvig et al., 2018; Weimer et al., 2009), GSK3 (López-Tobón et al., 2019; Yokota et al., 2010), Arp2/3 (Pinyol et al., 2007; Wang et al., 2016), adducins (Kalebic et al., 2019), PALMD (Kalebic et al., 2019) and others. All these molecules influence cell polarity via induction of new processes, which is particularly relevant in the context of flexibility of cell polarity.

#### Cell-extrinsic factors

Several microenvironmental factors, such as components of the extracellular matrix (ECM), secreted proteins and molecules mediating cell-cell interactions have been identified as cell extrinsic regulators of neuronal polarization (Hansen et al., 2017; Namba et al., 2015). Similar molecules might easily be involved in progenitor polarization. When studying process growth in both progenitors and neurons, the fundamental question is how to establish specificity between various processes. Whereas both bIPs and bRG can grow short processes or lamellate expansions that serve as platforms for receiving signals from the local environment (Kalebic et al., 2019; Rakic, 1972; Reillo et al., 2017; Schmechel and Rakic, 1979), only bRG can grow a long basal process that allows them to contact and receive signals from the basal lamina (Kalebic and Huttner, 2020). Similarly, neurons also grow two types of processes with distinct functions; leading and trailing processes for migration, as well as axon and dendrites for development of neural circuitry. To establish a process with specific identity, neurons use microenvironmental cues, such as secreted factors and cell-cell interactions (Namba et al., 2015).

Multipolar neurons in mouse embryonic neocortex can be polarized in two different ways. The first way is to extend a trailing process (future axon) (Hatanaka and Yamauchi, 2013; Namba et al., 2014) before the formation of the leading process by a mechanism known as ‘touch and go’, which consists of an interaction of a minor process of the multipolar neuron with the pioneering axons of the early-born neurons via TAG-1 (Namba et al., 2014). The second way is to establish a leading process and subsequently the trailing process (Nakamuta et al., 2011; Sakakibara et al., 2014), which occurs through the stabilization of the leading process by an N-cadherin interaction with the radial glia (Jossin and Cooper, 2011; Kawauchi et al., 2010; Xu et al., 2015). Considering that TAG-1 is also involved in maintaining the basal process of aRG (Okamoto et al., 2013), it is important to examine whether similar molecules and mechanisms are involved in establishing the identity of the basal process of bRG.

Most of our knowledge about the growth of the progenitors’ basal process comes from studies on aRG, although many aspects have been shown to be true for the bRG. Proper anchoring of the basal endfoot to the basal lamina requires various secreted ECM components, such as collagens and laminins, as well as their receptors on the basal process, such as GPR56 (Bae et al., 2014) and various subunits of integrins (Fietz et al., 2010). ECM components are also implicated in the inheritance of polarity from progenitor cells to neurons in vertebrate retina (Randlett et al., 2011). Retinal neuroepithelial cells require a contact to the basal membrane via ECM components, such as laminin, in order to maintain their cell polarity, which in turn is important for the subsequent inheritance of polarity for the newborn neuron (Randlett et al., 2011).

### Morphological polarity

There are two examples of how neurons could inherit morphological polarity (see Glossary, Box 1) from their mother cells: (1) in gyrencephalic species, such as the macaque, the majority of neurons are derived from morphologically polarized bRG (Betizeau et al., 2013), allowing the possibility of inheritance of their ‘pseudoapicobasal polarity’ and its transformation into the front-rear neuronal polarity (Fig. 2C); and (2) in the vertebrate retina, the basal process of neuroepithelial cells acts as a signaling center for maintaining the progenitor cell morphological polarity (Randlett et al., 2011) that, when inherited by the daughter neuron, serves as a basis for its axon-dendrite polarity.

The length of specific processes is a major feature of morphological polarity because it contributes to determining the process identity. To establish proper axon-dendrite polarity, neurons need to extend one process rapidly as an axon and suppress the elongation of the remaining neurites. To this end, neurons use three different mechanisms (Namba et al., 2015). First, the length of the nascent axon itself helps the accumulation of the polarity proteins at the tip of the growing process (Naoki et al., 2011). Second, the positive feedback loop at the tip of growing axon, which involves PI3K and Rac1 (Nishimura et al., 2005). Third, the negative feedback signal from the growing process to the minor processes, which can for example involve PKA, CaMKI and RhoA (Shelly et al., 2010; Takano et al., 2017).

In species with an expanded neocortex, both BPs (Kalebic and Huttner, 2020) and neurons (Gertz and Kriegstein, 2015) exhibit a remarkable flexibility of their morphological polarity (Fig. 3; Table 1). Conversely, in species with a small neocortex, BPs and neurons do not exhibit significant flexibility of their morphological polarity (Kalebic and Huttner, 2020; Namba et al., 2015) (Table 1). Similarly, aRG in all examined mammals have a rather non-flexible morphological polarity, with their morphological dynamics mainly limited to growth cone-like endfeet and filopodia-like protrusions (Fujita et al., 2020; Kalebic et al., 2019; Kosodo et al., 2004; Nakagawa et al., 2019; Reillo et al., 2017; Yokota et al., 2010) (Table 1).

**Fig. 5.**
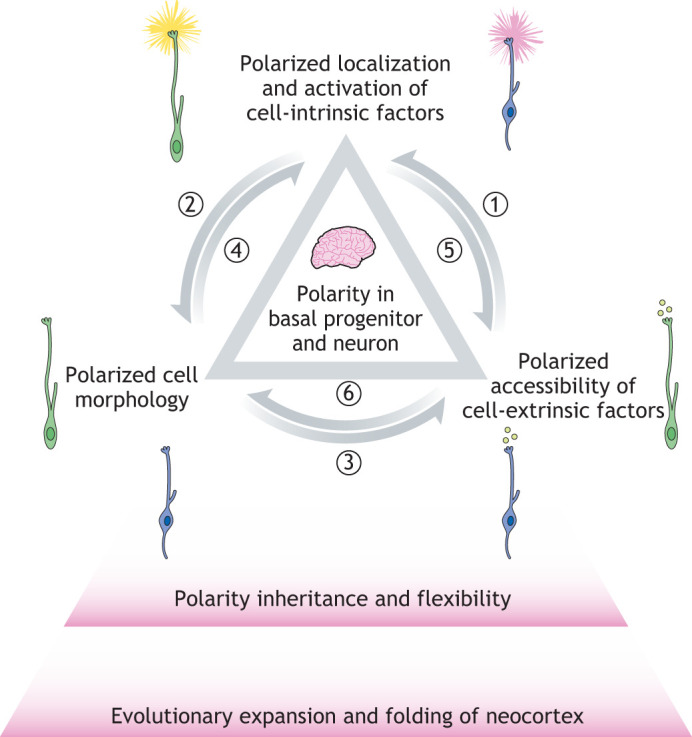
**Inheritance and flexibility of cell polarity in neocortex expansion and folding.** Polarized localization and activation of cell-intrinsic factors is the principal molecular determinant of cell polarity (upper). Polarized accessibility of cell-extrinsic factors (right) can influence the polarized localization and activity of cell-intrinsic factors (1), which in turn can act as determinants of morphological cell polarity (2). Changes in cell morphology (left) can be responsible for both polarized exposure to new extracellular factors (3) and localization of cell-intrinsic factors (4). Cell polarization induced by cell-intrinsic factors will change the accessibility of different extracellular signals (5), which in turn leads to further changes in morphological cell polarity (6). Such triangular regulation of cell polarity inheritance and flexibility in basal progenitors and neurons enables the evolutionary expansion and folding of the neocortex.

In conclusion, the inheritance and flexibility of cell polarity are regulated through the triangular interplay of cell morphology with intrinsic and extrinsic molecular factors (Fig. 5). Polarized localization and activation of cell-intrinsic factors is the principal molecular determinant of cell polarity. Cell-extrinsic factors can influence the localization and activity of cell-intrinsic factors and thereby act as determinants of cell polarity. Moreover, changes in cell morphology can be responsible for both polarized localization of cell-intrinsic factors and exposure to new extracellular factors. Finally, cell polarization induced by cell-intrinsic factors materializes itself through changes in cell morphology and consequently exposure to different extracellular signals.

## Future perspectives

Neocortex expansion occurred in both the radial and tangential axis. The tangential expansion has been many-fold greater on the basal side than on the apical side, which led to the characteristic gyrification pattern as a way to accommodate additional neurons (Rakic, 2009). It is widely accepted that the increased proliferative capacity of BPs, and bRG in particular, is the key requirement for the evolutionary expansion of the neocortex, because it underlies the increase in the production of projection neurons (Kalebic and Huttner, 2020; Llinares-Benadero and Borrell, 2019; Lui et al., 2011; Namba and Huttner, 2017; Rakic, 2009; Sun and Hevner, 2014), as well as interneurons (Hladnik et al., 2014) and astrocytes (Rash et al., 2019).

Here, we have hypothesized that the inheritance and flexibility of cell polarity underlie the evolutionary expansion of the human neocortex by promoting amplification of neural progenitors and tangential dispersion of neurons; two key prerequisites for cortical folding. It might appear to be counterintuitive that the flexibility of inherited cell polarity, and therefore the loss of inherited polarity, synergistically contributes to the evolutionary expansion of the neocortex. The sequentiality of these cellular events likely plays a key role. For example, a daughter bRG that inherits the basal process from the mother bRG maintains its proliferative capacity due the inherited basal process. Its own daughter bRG that did not inherit the basal process will, by virtue of the flexibility of its cell polarity, be able to regrow one. This regrowth will, in turn, allow it to maintain the bRG identity and the proliferative capacity (Fig. 2C).

The molecular mechanisms that underlie the flexibility in bRG polarity are not yet known. However, it is possible that the same molecular players known to regulate cell polarity of aRG and neurons are also active in bRG. Therefore, polarized localization or activity of members of the Par complex, cytoskeleton components and modifiers, mRNAs and RNA-binding proteins might play an evolutionary role in the flexibility of cell polarity. Members of the Par complex are interesting candidates to examine as they show polarized localization in progenitors and neurons, and are crucial for transmitting extracellular signals to morphological regulators, such as small GTPases (Namba et al., 2015). In addition, the Par complex can regulate transcription through the Hippo pathway (Zhang et al., 2016), which has been implicated in the evolutionary expansion of the neocortex (Kostic et al., 2019). As dynamic morphological changes require active supply of the plasma membrane components, cell metabolism needs to fulfill the demand of membrane lipids and proteins. Therefore, the specialization to the anabolic metabolism, which has been hypothesized to play a role in human brain evolution (Namba et al., 2021), could provide a basis for process growth, allowing for the flexibility of cell polarity. A comprehensive identification of both cell intrinsic and cell extrinsic molecular regulators of polarity flexibility is required in both bRG and neurons. Furthermore, the inheritance of cell polarity between bRG and neurons should be further experimentally examined. Finally, it is important to understand which mechanisms in migrating neurons underlie the loss of flexibility in cell polarity and the establishment of the stable axon-dendrite polarity.

Disruptions of cell polarity of progenitors and neurons can cause severe diseases, such as neocortical malformations (Juric-Sekhar and Hevner, 2019; Klingler et al., 2021; Romero et al., 2018). Lissencephaly is a cortical malformation characterized by smooth cerebral surface and impaired neuronal migration (Klingler et al., 2021; Reiner et al., 1993). LIS1 is microtubule-associated protein that is often found mutated in human lissencephaly (Reiner et al., 1993). Interestingly, the disruption of LIS1 in mouse neocortical neurons resulted in a failure to establish the front-rear polarity (Youn et al., 2009). It is, therefore, important to examine whether an impairment of cell polarity inheritance and flexibility – in both bRG and neurons – could be causative to other human neocortical malformations.

Recent technological advances that enable the use of emerging *in vitro* and animal model systems will likely play a key role in testing the hypothesis that has been put forth here. Gyrencephalic animal models, like ferret and macaque, are helpful because they allow the study of progenitor cell biology and neuronal migration in a physiological environment of a folded neocortex (Betizeau et al., 2013; Gertz and Kriegstein, 2015; Gilardi and Kalebic, 2021; Llinares-Benadero and Borrell, 2019). Nevertheless, certain neurodevelopmental features, including those underlying various neurodevelopmental disorders, are characteristic to humans. In this context, human cerebral organoids have an instrumental role as they allow analysis of the development of the human brain carrying pathogenic mutations (Chiaradia and Lancaster, 2020; Sidhaye and Knoblich, 2021). Future technological improvements in organoid protocols, likely focusing on advancements in recapitulating neuronal migration, will be valuable to examine the sequence of polarization events in human neurons. Furthermore, in order to link the morphological and molecular diversities of newborn neurons and progenitors, various single cell ‘-omics’ approaches will benefit from taking into consideration the polarity features. Combined with a manipulation of cell polarity with a high spatio-temporal resolution, these approaches will pave the road for illuminating the relevance of polarity flexibility and inheritance for brain development.
